# An Investigation into the Relationship among Psychiatric, Demographic and Socio-Economic Variables with Bayesian Network Modeling

**DOI:** 10.3390/e20030189

**Published:** 2018-03-12

**Authors:** Gunal Bilek, Filiz Karaman

**Affiliations:** 1 Department of Statistics, Bitlis Eren University, 13000 Bitlis, Turkey; 2 Department of Statistics, Yildiz Technical University, 34349 Istanbul, Turkey

**Keywords:** Bayesian networks, bnlearn, data discretization, Beck Depression Inventory, Beck Hopelessness Scale, Rosenberg Self-Esteem Scale

## Abstract

The aim of this paper is to investigate the factors influencing the Beck Depression Inventory score, the Beck Hopelessness Scale score and the Rosenberg Self-Esteem score and the relationships among the psychiatric, demographic and socio-economic variables with Bayesian network modeling. The data of 823 university students consist of 21 continuous and discrete relevant psychiatric, demographic and socio-economic variables. After the discretization of the continuous variables by two approaches, two Bayesian networks models are constructed using the bnlearn package in R, and the results are presented via figures and probabilities. One of the most significant results is that in the first Bayesian network model, the gender of the students influences the level of depression, with female students being more depressive. In the second model, social activity directly influences the level of depression. In each model, depression influences both the level of hopelessness and self-esteem in students; additionally, as the level of depression increases, the level of hopelessness increases, but the level of self-esteem drops.

## 1. Introduction

Bayesian networks (BNs), also called belief networks, are graphical structures used to reason and represent knowledge in an uncertain field. They are a combination of graph theory and probability theory to explore relationships between variables [1]. The network structure is a directed acyclic graph (DAG) in which nodes represent random variables [2,3] and arcs represent direct dependencies between variables [4,5].

In psychiatry, scales are used as a helper tool to be used in diagnosis. In this study, the Beck Depression Inventory (BDI), the Beck Hopelessness Scale (BHS) and the Rosenberg Self-Esteem Scale (RSES) are used to investigate the psychiatric characteristics of university students. Bayesian networks have been widely used in many domains including engineering [6], customer behaviors [7,8,9], social behaviors [10,11], clinical decision support [12], system biology [13,14], ecology [15], and so on.

In this paper, the psychiatric variables, which are continuous, are discretized using two approaches: (i) expert knowledge in literature and (ii) a statistical method. Then, Bayesian networks are used to analyze the relationship among BDI, BHS, RSES, demographic variables and socio-economic variables.

The structure of this paper is as follows: In Section 2, the data used in this study are briefly described. In Section 3, the theory of Bayesian networks is summarized. In Section 4, the continuous data are discretized using two approaches. In Section 5, the data are analyzed, and the results are interpreted. Finally, in Section 6, the two methods are compared, and the study is concluded.

## 2. Data

In this study, the data are composed of three types of variables: (i) psychiatric variables, (ii) demographic variables and (iii) socio-economic variables.

### 2.1. Psychiatric Variables

#### 2.1.1. Beck Depression Inventory

The Beck Depression Inventory (BDI), created by [16], is a self-report inventory used to measure the severity of depression in individuals aged 13 and over. The BDI consists of 21 multiple-choice questions, with zero being the lowest score and three being the highest score in each question. The total BDI score is calculated by summing the answers of the 21 questions. Possible BDI scores range from 0–63.

#### 2.1.2. Beck Hopelessness Scale

The Beck Hopelessness Scale (BHS), developed by [17], is a self-report inventory that shows the level of hopelessness in adults, aged 17–80. It consists of 20 true-false questions. Eleven items are keyed true and nine false. Therefore, the answers are summed up, and the total BHS score is calculated. BHS scores range from 0–20.

#### 2.1.3. Rosenberg Self-Esteem Scale

The Rosenberg Self-Esteem Scale (RSES), created by [18], is a self-report inventory that represents how an individual judges his or her self-worth. The RSES consists of 10 items and is rated on a four-point Likert scale with one, strongly disagree, and three, strongly agree, for the 1st, 2nd, 4th, 6th and 7th items and one, strongly agree, and three, strongly disagree, for the 3rd, 5th, 8th, 9th and 10th items. The RSES scores are calculated by summing the answers of the 10 items, and they lay in the interval 0–30.

### 2.2. Participants

The participants of this study are the students of Bitlis Eren University, located in Turkey, in the 2016–2017 academic year. In the first week of May 2017, the students were given a four-page form consisting of the Turkish versions of the Beck Depression Inventory, the Beck Hopelessness Inventory, the Rosenberg Self-Esteem Scale and demographic and socio-economic variables. The students were asked to fill the forms out on a voluntary basis. A total of 890 students from five schools of Bitlis Even University were included in the study, and the data of 823 students were obtained after extracting the missing data.

### 2.3. Demographic Variables

There are only two demographic variables in the data, which are Gender and Age.

### 2.4. Socio-Economic Variables

There are 16 socio-demographic variables in the data. These variables are as follows: School_type, Smoking_status, Type_of_accommodation, Alcohol_use, Social_activity, $Student^{\prime }s\_job$, Family_structure, $Father^{\prime }s\_educational\_status$, Family_income, $Mother^{\prime }s\_educational\_status$, $Student^{\prime }s\_income$, Number_of_siblings, Type_of_settlement, $Father^{\prime }s\_occupation$, $Mother^{\prime }s\_occupation$ and Relationship_status. Both demographic and socio-economic variables are described in Table 1.

## 3. Bayesian Networks

Let G=(N,A) be a directed acyclic graph (DAG), where **N** is a finite set of nodes and **A** is a finite set of arrows between the nodes. The DAG characterizes the structure of the BN.

Each node *n*∈**N** in the graph **G** represents a random variable $X_{n}$. The set of variables associated with the graph **G** is denoted by $X={\left(X_{n}\right)}_{n\in N}$. A local probability distribution ($p(x_{n}|x_{pa(n)})$) is allocated to every node with parents pa(n). A BN for random variables **X** is a combination of **G** and **P** (G,P), where **P** is the set of local distributions for all variables in the network [19].

The absence of arrows in **G** encodes conditional independence between the random variables **X** through the factorization of the joint probability distribution,

(1) $$p\left(x\right)=\prod_{n\in N}p\left(x_{v}|x_{pa(v)}\right).$$

In Equation (1), there are both discrete and continuous variables as shown in [20]. **N** consists of Ω and Ψ (N=Ω⋃Ψ), where Ω and Ψ represent the sets of discrete and continuous variables, respectively. The random variables **X** can be redefined as $X={\left(X_{n}\right)}_{n\in N}=\left(I,J\right)=\left({\left(I_{\omega }\right)}_{\omega \in \Omega },{\left(J_{\psi }\right)}_{\psi \in \Psi }\right)$, where **I** and **J** are the sets of discrete and continuous variables, respectively. To ensure the availability of exact local computation methods, discrete variables are not allowed to have continuous parents. The joint probability distribution then factorizes into a discrete part and a mixed part [21],

(2) $$p\left(x\right)=p\left(i,j\right)=\prod_{\omega \in \Omega }p\left(i_{\omega }|i_{pa(\omega )}\right)\prod_{\psi \in \Psi }\left(j_{\psi }|i_{pa(\psi )},j_{pa(\psi )}\right).$$

### 3.1. Structural Learning

There are two kinds of structure learning algorithms. The first is the search-and-score algorithm, which allocates a score to every Bayesian network structure and chooses the structure model with the highest score. The latter is called constraint-based structural learning, and it creates a set of conditional independence analysis on the data and uses this analysis to produce an undirected graph. With an additional independence test, the network is converted into a Bayesian network [22]. In this study, the tabu search algorithm [23] is preferred.

### 3.2. Parameter Learning

After learning the structure of the BN from the data, the parameters can be estimated and updated. There are two approaches widely-used in the literature: (i) maximum likelihood estimation and (ii) Bayesian estimation [24]. In this paper, Bayesian estimation based on Dirichlet priors is used.

## 4. Data Discretization

The data discretization is a technique that converts continuous data into discrete data with a finite number of intervals, and it has become remarkably popular in many research areas including data mining, machine learning, artificial intelligence and Bayesian networks. There are many reasons to discretize data, among which the most important ones are (i) reducing and simplifying the dataset, (ii) making modeling fast and easy, (iii) obtaining easily interpretable outputs [25] and (iv) the statistical method to be used may operate in discrete data only as in this study.

**Figure 1:**
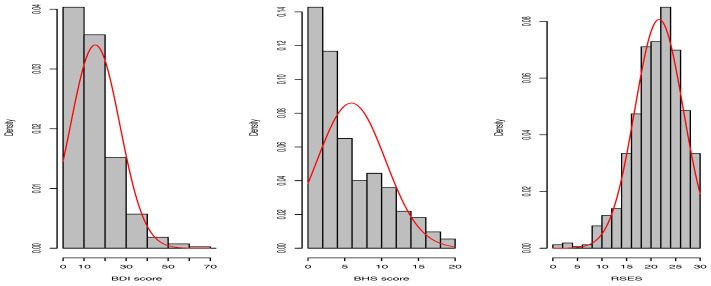
Histograms of psychiatric variables. BDI, Beck Depression Inventory; BHS, Beck Hopelessness Scale; RSES, Rosenberg Self-Esteem Scale.

As Figure 1 shows, the psychiatric variables are not normally distributed, which violates the Gaussian Bayesian networks (GBNs) assumption. In this case, we may specify a suitable conditional distribution for every variable and build a hybrid Bayesian network [24]. Nevertheless, this approach requires prior knowledge, which is not available in our case. Therefore, we transform the continuous variables into discrete ones, i.e., discretization, and build discrete Bayesian networks. In this paper, we discretize the continuous variables in two ways, which are given in the next two sections.

### 4.1. Data Discretization by Expert Knowledge

One way to discretize continuous variables is to categorize them by following the studies in the literature. The intervals for the continuous variables in this study were categorized by the domain experts in the previous studies. Therefore, we use this knowledge to discretize the psychiatric variables. Hence, the discretization of the psychiatric variables is as follows:

1. Beck Depression Inventory Score: We discretize this variable following [26] into four levels: BDI scores 0–10 show none or minimal depression, 10–18 mild depression, 19–29 moderate depression and 30–63 severe depression.
2. Beck Hopelessness Scale Score: We discretize this variable following [27] into four levels: The BHS scores 0–3 correspond to none or minimal hopelessness, 4–8 to mild hopelessness, 9–14 to moderate hopelessness and 15–20 to severe hopelessness.
3. Rosenberg Self-Esteem Scale Score: We discretize this variable following [28] into three levels: The RSES scores below 15 indicate low self-esteem, 15–25 normal self-esteem and 26–30 high self-esteem.

### 4.2. Data Discretization by Statistical Methods

When no prior knowledge from domain experts is available, discretization methods are used to discretize the continuous variables. In the literature, there exist many discretization methods. Some of them are entropy-based discretization [29], error-based discretization [30], the one-rule discretizer (1RD) [31], equal frequency discretization [32] and information-preserving discretisation [33].

In this paper, the information-preserving discretization method is used to discretize the continuous variables. In this method, the basic notion is to initially discretize each variable into large numbers of intervals, say $k_{1}$. Hence, the amount of information lost is kept minimum. Then, the algorithm repeats over the variables and retains, for each of them, the pair of attached intervals minimizing the loss of pairwise mutual information. Once all variables have $k_{2}$, which is the number of intervals the user specifies, ≤$k_{1}$ intervals left, the algorithm stops [24]. One of the reasons to use this method in this paper is that it enables us to specify the number of intervals. As the continuous variables in our study measure the severity of depression, hopelessness and self-esteem, it is reasonable to discretize them into three categories as low, normal and high. Another reason is that this method can be easily applied in R.

When we discretize the psychiatric variables using the discretize function in R (see Appendix A), the intervals are obtained as follows:

1. Beck Depression Inventory Score:
  - (0, 11.5]      : low
  - (11.5, 20.8] : normal
  - (20.8, 63]    : high
2. Beck Hopelessness Scale Score:
  - (0, 6.89]     : low
  - (6.89, 13]   : normal
  - (13, 20]      : high
3. Rosenberg Self-Esteem Score:
  - (0, 15.8]       : low
  - (15.8, 19.8] : normal
  - (19.8, 30]    : high

## 5. Analysis

In this section, we analyze the datasets by constructing suitable BNs. All of the analysis is conducted in statistical software R using bnlearn [34], lattice [35], gRain [36] and Rgraphviz [37] packages.

First of all, to test the reliability of the psychiatric scales, Cronbach’s alpha internal consistency coefficients [38] need checking. Cronbach’s alpha internal consistency coefficients for BDI, BHS and RSES are 0.89, 0.87 and 0.83, respectively, meaning these scales are safe (greater than 0.70) for use in the analysis.

We build two different discrete BNs using the same method as two different approaches of discretization were used. We call the BN whose data were discretized by expert knowledge Bayesian Network 1 (BN1) and call the BN whose data were discretized by Information-Preserving Discretization Bayesian Network 2 (BN2).

### 5.1. Bayesian Network 1

We build the BN using R, and all the code used is given in Appendix B. Before learning the BN, we must specify a prior BN. Since we have no prior knowledge available, we specify an empty DAG. In addition, we must block all the arrows towards Gender and Age since no other variables can affect these two variables.

**Figure 2:**
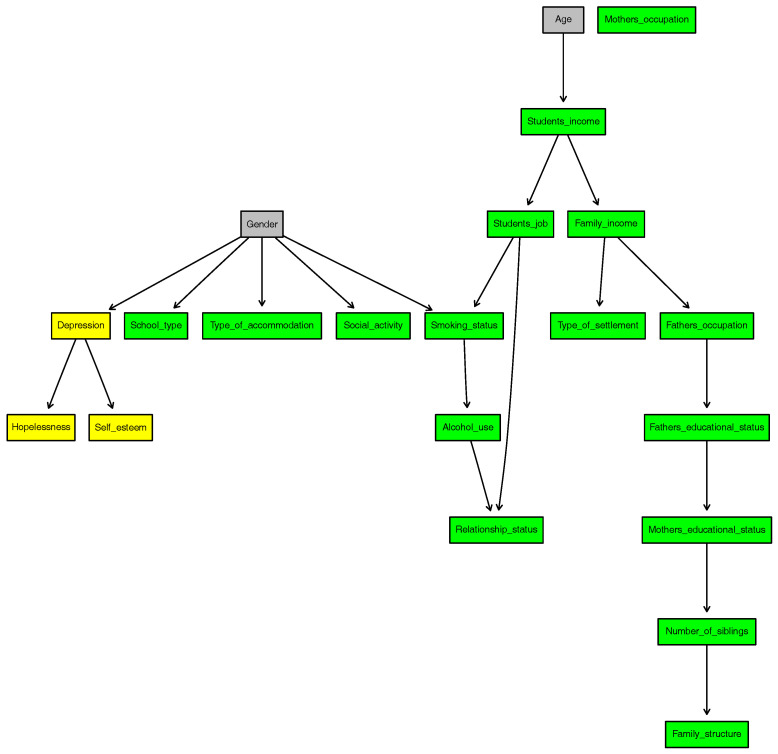
DAG of BN1.

Figure 2 shows the DAG of the BN1 and the network score of BN1 is calculated as −15,214.8.

To summarize the significant results, $Mother^{\prime }s\_occupation$ is independent of all variables. This is because 93% of the mothers are unemployed.

Age and Gender are independent of all variables as we specified in the model. However, Age directly affects $Student^{\prime }s\_income$, and Gender directly affects Depression, School_type, Type_of_accommodation, Social_activity and Smoking_status.

While Depression is dependent on Gender, the other two psychiatric variables Hopelessness and Self_esteem are conditionally independent of all demographic and socio-economic variables (excluding $Mother^{\prime }s\_occupation$) given Depression. However, Hopelessness and Self_esteem are directly dependent on Depression.

**Figure 3:**
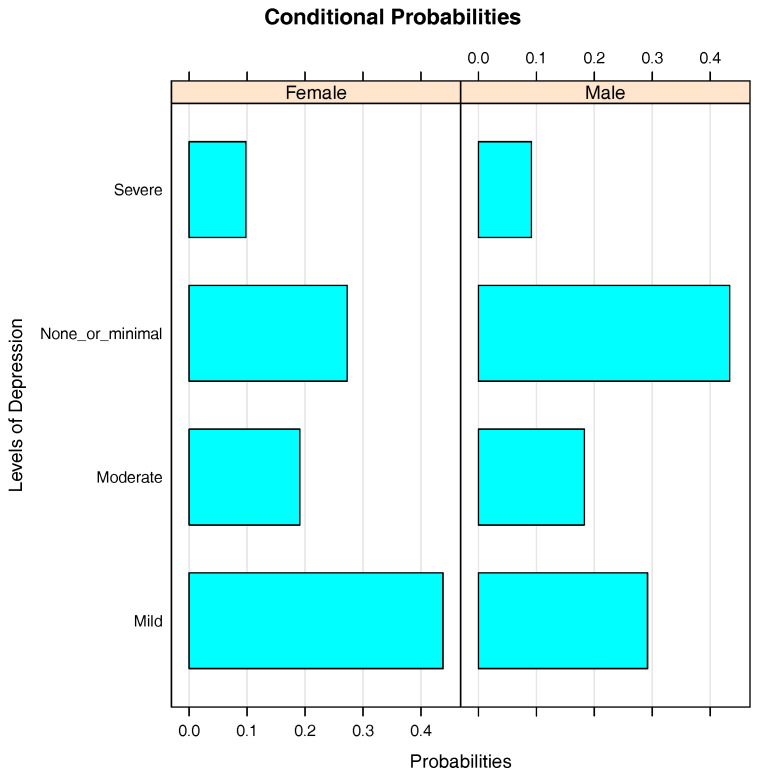
Bar chart of conditional probabilities of Depression given Gender.

Figure 3 represents the conditional probabilities of Depression given Gender, i.e., P(Depression|Gender). It is clear that female and male students have approximately equal probabilities of having severe depression (10%) and moderate depression (19%). While the probability of a male student having none or minimal depression is 43%, it is 27% for his female counterpart. However, female students are more likely to have mild depression (44%) than male students (29%). From Figure 3, it can be said that female students are more likely to have a higher level of depression than male students.

**Figure 4:**
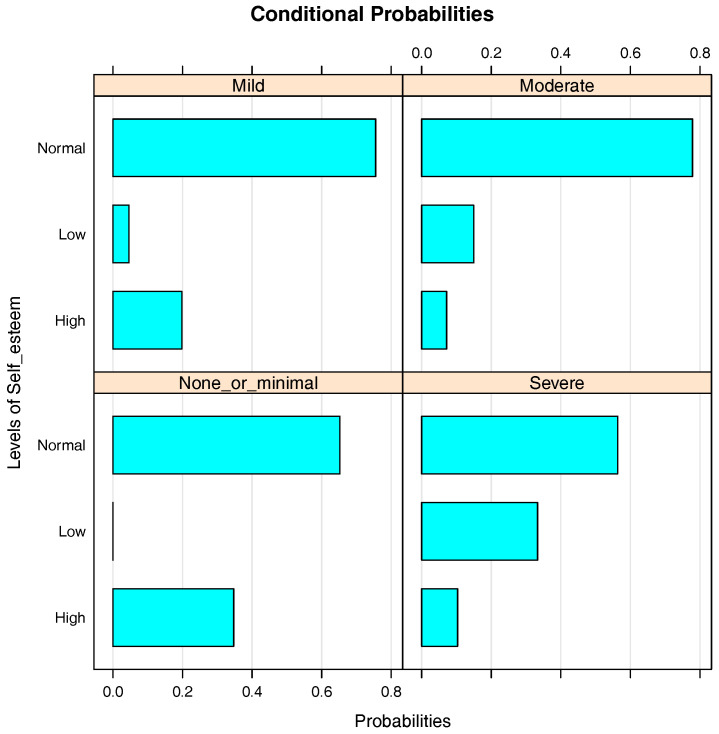
Bar chart of the conditional probabilities of Self_esteem given Depression.

Figure 4 shows the bar charts of the conditional probabilities of Self_esteem given Depression. As the level of depression increases from none or minimal to severe depression, the probability of having a low level of self-esteem increases. For the depression levels none or minimal, mild, moderate and severe depression, the probabilities of students having low self-esteem are 0.01, 0.04, 0.15 and 0.33, respectively, while the probabilities of having high self-esteem are 0.35, 0.20, 0.08 and 0.11, respectively. Therefore, it can be concluded that the level of depression has a negative influence on self-esteem.

**Figure 5:**
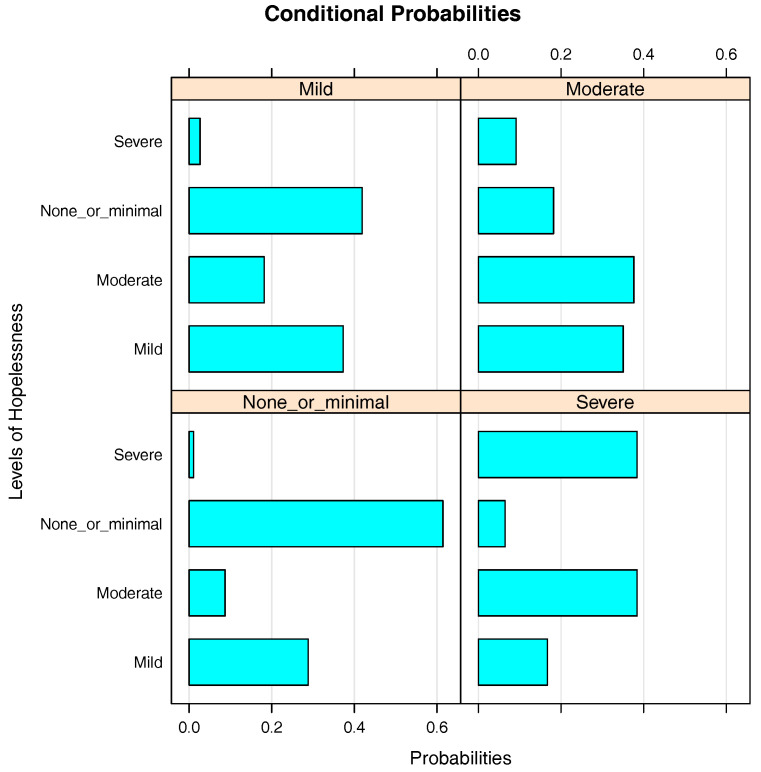
Bar chart of the conditional probabilities of Hopelessness given Depression.

Figure 5 presents the bar chart of the conditional probabilities of Hopelessness given Depression. Unlike with Self_esteem, generally speaking, there is a positive relationship between Hopelessness and Depression. As the level of depression in students increases, so does the probability of having a higher level of hopelessness. The probabilities of having none or minimal hopelessness, mild hopelessness, moderate hopelessness and severe hopeless are respectively 0.61, 0.29, 0.09 and 0.01 in students with none or minimal depression, 0.42, 0.37, 0.18 and 0.03 in students with mild depression, 0.18, 0.35, 0.37 and 0.10 in students with moderate depression and 0.07, 0.17, 0.38 and 0.38 in students with severe depression. These figures show that a higher level of depression in students results in a higher level of hopelessness.

### 5.2. Bayesian Network 2

Now, we build a BN just like we did in the previous section. Again, we specify an empty DAG as the prior DAG and block all the arrows towards Age and Gender (see Appendix C).

**Figure 6:**
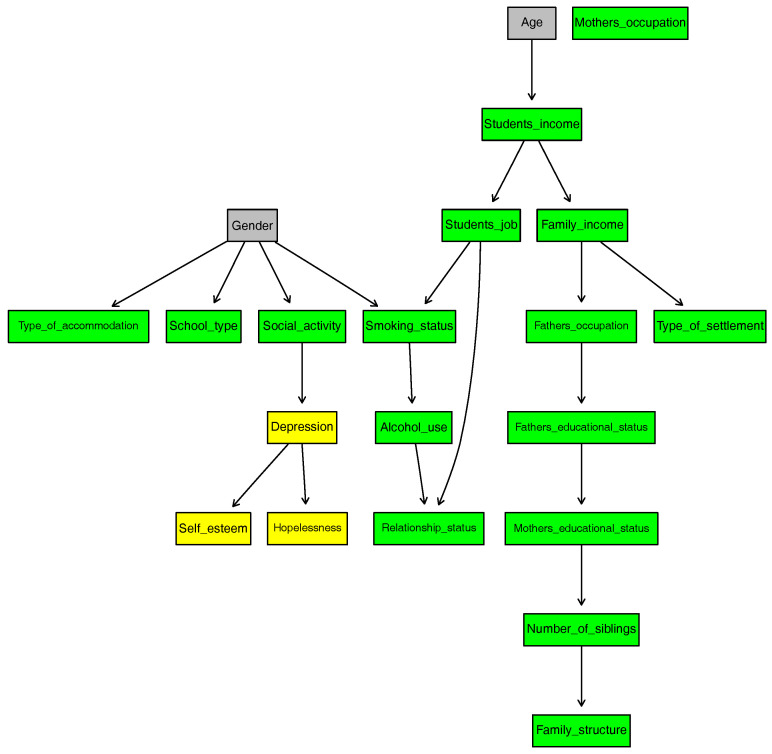
DAG of BN2.

Figure 6 represents the DAG of BN2, and the network score of BN2 is −14,748.93.

To abstract the notable relationships, Age directly affects $Student^{\prime }s\_income$, while Gender directly affects Type_of_accommodation, School_type, Social_activity and Smoking_status.

Of all the demographic and socio-economic variables, the only variable that has a direct influence on one of the psychiatric variables, that is Depression, is Social_activity. In addition, Self_esteem and Hopelessness are directly dependent on Depression, but both of them are conditionally independent of all the demographic and socioeconomic variables (excluding $Mother^{\prime }s\_occupation$) when the status of Depression is known.

**Figure 7:**
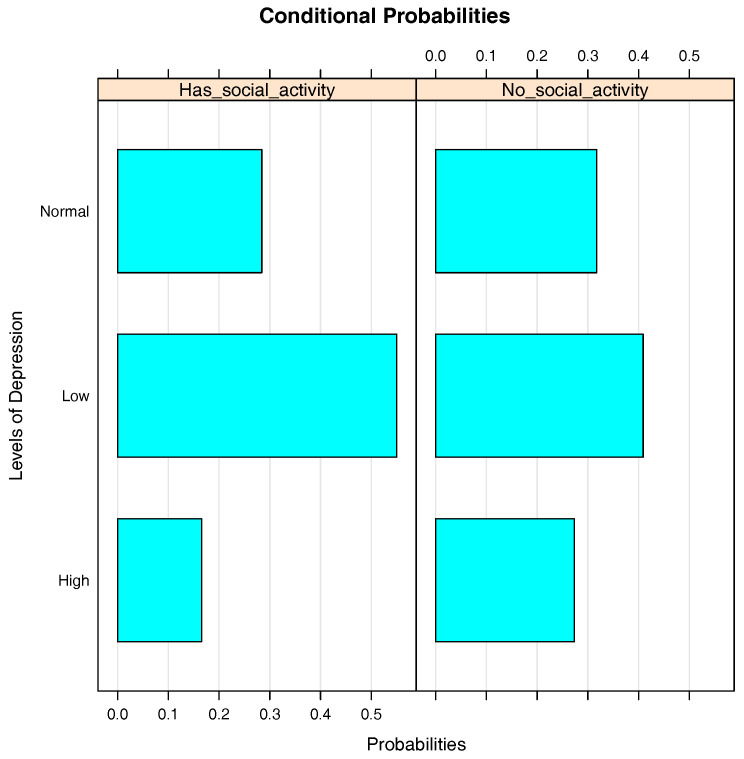
Bar chart of the conditional probabilities of Depression given Social_activity.

Figure 7 is the bar chart of the conditional probabilities of Depression given Social_activity. It shows that the presence of Social_activity has a negative impact on Depression. While only approximately 17% of the students with social activity have a high level of depression, this proportion is about 28% in the students with no social activity. In addition, the proportion of students with a low level of depression is significantly lower in the students with social activity compared to those with no social activity.

**Figure 8:**
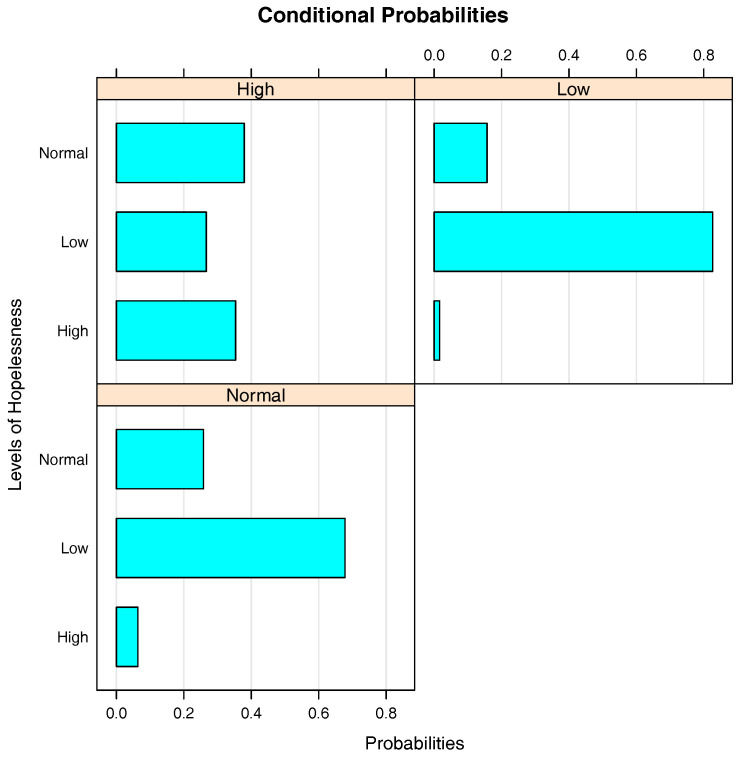
Bar chart of the conditional probabilities of Hopelessness given Depression.

Figure 8 is the bar chart of the conditional probabilities of Hopelessness given Depression. When a student is known to have a low level of depression, the probability that he/she has a low level of hopelessness is around 0.83, while the probability of his/her having a high level of hopelessness is merely 0.02. In the students with high level of depression, the proportions of students with low and high levels of hopelessness are approximately 0.27 and 0.35, respectively. Therefore, it is concluded that there is a positive correlation between Depression and Hopelessness.

**Figure 9:**
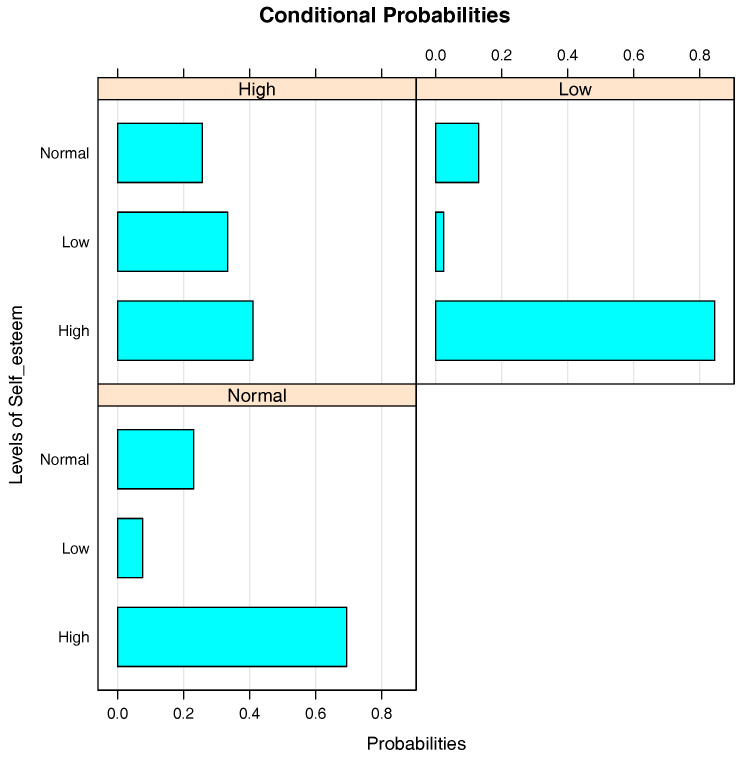
Bar chart of the conditional probabilities of Self_esteem given Depression.

Figure 9 illustrates the conditional probabilities of Self_esteem given Depression. At first glance, it is noted that the students who have a high level of self-esteem dominate the others in each group. The probabilities that a student has high and low levels of self-esteem are about 85% and 2%, respectively, when he/she has a low level of depression, whereas these probabilities are 41% and 33%, respectively, when the student has a high level of depression. These numbers indicate that as the severity of Depression increases, the severity of self-esteem decreases.

## 6. Discussion and Conclusions

In this paper, the relationship among the 3 psychiatric, 2 demographic and 16 socio-economic variables was investigated through BN modeling in R. The data were obtained from 823 university students via a survey.

In the analysis, firstly, the Beck Depression Inventory, the Beck Hopelessness Scale and the Rosenberg Self-Esteem Scale scores were calculated from the answers of students. Then, Cronbach’s alpha internal consistency coefficients for the these psychiatric scales were tested and found satisfying. Next, the continuous data were discretized by two approaches, which led to two BN models (BN1 and BN2). Finally, after building the BNs with the tabu search algorithm, inference and queries were made through graphs and conditional probabilities.

To summarize the results, only the occupational status of the mother has no relation with the other variables in both models.

According to BN1, the gender of the students directly influences the choices of students on school type, accommodation type, social activity and smoking status, while the age directly influences only the income of the student. The gender also directly affects the level of depression of students, and female students have a higher chance of being more depressive than male students. This finding is supported by similar studies [39,40,41,42].

According to BN2, similar to BN1, only the income of the students is directly dependent on the age. The gender has direct influence on accommodation type, school type, social activity and smoking status. In addition, depression is directly dependent on social activity. The presence of social activity has a negative effect on depression, which is also supported by [43,44]. Furthermore, self-esteem and hopelessness are directly dependent on depression.

To compare BN1 and BN2, firstly, BN2’s network score is greater than that of BN1, meaning BN2 is more likely to produce better predictions than BN1. While depression is directly dependent on the gender in BN1, it is conditionally independent of the gender given social activity in BN2. In each model, hopelessness and self-esteem are directly dependent on depression and conditionally independent of all the demographic and socio-economic variables excluding the occupational status of the mother, which is independent of all the variables.

In both models, one of the most important results is that as the level of depression rises, so does the level of hopelessness. This finding is consistent with other studies in the literature [45,46]. Another one is that, in contrast to hopelessness, the rise in the severity of depression results in lower self-esteem scores in students. This finding is in line with other studies on the relationship between depression and self-esteem [43,47,48].

## Figures and Tables

**Table 1:** Demographic and socio-economic data description.

Variable	Description	State Description
Age	Age of student	20−, 20–23, 23+
Gender	Gender of student	Male, female
Family_structure	Family structure of the student	Nuclear, extended, single parent, mother or father died
$Father^{\prime }s\_educational\_status$	Educational level of student’s father	Illiterate, primary school, secondary school, high school, university, Master’s or PhD
$Mother^{\prime }s\_educational\_status$	Educational level of student’s mother	Illiterate, primary school, secondary school, high school, university, Master’s or PhD
Family_income	Student’s family’s monthly income in TL*	0–1400TL, 1401–3000TL, 3001–5000TL, 5000 + TL
$Student^{\prime }s\_income$	Student’s monthly income in TL*	0–500TL, 501–750TL, 751–1000TL, 1000 + TL
Number_of_siblings	Number of siblings student has	1, 2, 3, 4, 5+
Type_of_settlement	Type of settlement students were born in	Village or town, district, city, metropolis
$Father^{\prime }s\_occupation$	Occupation of student’s father	Unemployed, self-employed, public sector employee, private sector employee
$Mother^{\prime }s\_occupation$	Occupation of student’s mother	Unemployed, self-employed, public sector employee, private sector employee
School_type	Type of school student studying at	Faculty of Arts and Science, Faculty of Engineering, Faculty of Economics and Administrative sciences, Health School, Vocational School
Type_of_accommodation	Where student is living	Government dormitory, private dormitory, flat
Smoking_status	Whether or not student is a smoker	Smoker, not smoker
Alcohol_use	Whether or not student consumes alcohol	Alcohol user, not alcohol user
Social_activity	Whether or not student has any social activity	Has social activity, no social activity
$Student^{\prime }s\_job$	Whether or not student has a part-time or full-time job	Has job, no job
Relationship_status	Student’s relationship status	Single, in a relationship
TL: Turkish Lira (currency)		

## Appendix A. R Code for Section 4.2

#read and attach data

data <- read.table("data.txt",header=T)

attach(data)

#discretize continuous variables

d.frame <- data.frame(Self_esteem_score, BDI_score, BHS_score)

discrete.data <- discretize(d.frame, method = "hartemink",breaks = 3, ibreaks = 100,

       idisc = "interval")

## Appendix B. R Code for Section 5.1

#load required packages

library(bnlearn)

library(Rgraphviz)

library(gRain)

library(lattice)

#prepare data

data1 <- data.frame(Depression, Hopelessness, Self_esteem, Age, Gender,

         School_type,Family_structure, Fathers_educational_status,

         Mothers_educational_status,Family_income,Students_income,

         Number_of_siblings, Type_of_settlement,Fathers_occupation,

         Mothers_occupation,Type_of_accommodation, Smoking_status

         Alcohol_use, Students_job,Relationship_status,Social_activity)

#create ban list

banlist = matrix(c("Self_esteem","Age","Depression","Age","Hopelessness","Age",

      "School_type","Age","Family_structure","Age","Number_of_siblings",

      "Age","Fathers_educational_status","Age",

      "Mothers_educational_status", "Age","Family_income","Age",

      "Students_income","Age","Smoking_status","Age",

      "Type_of_settlement","Age","Fathers_occupation","Age",

      "Mothers_occupation","Age","Type_of_accommodation","Age",

      "Alcohol_use", "Age", "Students_job", "Age", "Relationship_status",

      "Age","Social_activity","Age","Self_esteem", "Gender","Depression",

       "Gender","Hopelessness","Gender","School_type","Gender",

      "Family_structure","Gender","Fathers_educational_status","Gender",

      "Mothers_educational_status", "Gender", "Family_income", "Gender",

      "Students_income","Gender","Number_of_siblings","Gender",

      "Type_of_settlement","Gender","Fathers_occupation","Gender",

      "Mothers_occupation","Gender","Type_of_accommodation","Gender",

      "Smoking_status","Gender","Alcohol_use","Gender","Students_job",

      "Gender","Relationship_status","Gender","Social_activity",

      "Gender","Age","Gender", "Gender","Age"), ncol = 2, byrow = TRUE)

#Build bayesian network and calculate network score

bn1 <- tabu(data1, blacklist = banlist)

fit1 <- bn.fit(bn1, data1, method="bayes")

score(bn1,data1)

#Plot Figure 2

highlight1 <- list(nodes = nodes(bn1),arcs = arcs(bn1),col="black")

g1 <- graphviz.plot(bn1, highlight = highlight1, shape="rectangle")

#Plot Figures 3, 4 and 5 respectively

bn.fit.barchart(fit1$Depression, ylab = "Levels of Depression")

bn.fit.barchart(fit1$Self_esteem, ylab = "Levels of Self_esteem")

bn.fit.barchart(fit1$Hopelessness, ylab = "Levels of Hopelessness")

## Appendix C. R Code for Section 5.2

#Create data frame by discretized intervals

Depression <- ifelse(discrete.data$BDI_score=="(0.938,11.5]","Low",

      ifelse(discrete.data$BDI_score=="(11.5,20.8]","Normal","High"))

Hopelessness <- ifelse(discrete.data$BHS_score=="(0.981,6.89]","Low",

      ifelse(discrete.data$BHS_score=="(6.89,13]" ,"Normal","High"))

Self_esteem <- ifelse(discrete.data$Self_esteem_score=="(0.971,15.8]","Low",

     ifelse(discrete.data$Self_esteem_score=="(15.8,19.8]","Normal","High"))

data2 <- data.frame(Depression, Hopelessness, Self_esteem, Age, Gender,

         School_type,Family_structure,Fathers_educational_status,

         Mothers_educational_status,Family_income,Students_income,

         Number_of_siblings, Type_of_settlement,Fathers_occupation,

         Mothers_occupation, Type_of_accommodation,Smoking_status,

         Alcohol_use, Students_job,Relationship_status,Social_activity)

#Build Bayesian network and calculate network score

bn2 <- tabu(data2, blacklist = banlist)

fit2 <- bn.fit(bn2, data2, method="bayes")

score(bn2, data2)

#Plot Figure 6

highlight2 <- list(nodes = nodes(bn2),arcs = arcs(bn2),col="black")

g2 <- graphviz.plot(bn2, highlight = highlight2, shape="rectangle")

#Plot Figures 7, 8 and 9 respectively

bn.fit.barchart(fit2$Depression, ylab = "Levels of Depression")

bn.fit.barchart(fit2$Self_esteem, ylab = "Levels of Self_esteem")

bn.fit.barchart(fit2$Hopelessness, ylab = "Levels of Hopelessness")
